# Supplementary material for: Single-cell transcriptomics following ischemic injury identifies a role for B2M in cardiac repair
Source: Commun Biol. 2021 Jan 29;4:146. doi: 10.1038/s42003-020-01636-3 (PMC7846780; doi:10.1038/s42003-020-01636-3)
Supplement: Supplementary file 2 — Supplemental Information [file 42003_2020_1636_MOESM2_ESM.pdf]

Supplementary information

Single-cell transcriptomics following ischemic injury identifies a role for B2M in cardiac repair

Bas Molenaar[1]#, Louk T. Timmer[1]#, Marjolein Droog[1], Ilaria Perini[1], Danielle Versteeg[1,2], Lieneke Kooijman[1], Jantine Monshouwer-Kloots[1], Hesther de Ruiter[1], Monika M. Gladka[1], and Eva van Rooij[1,2]*

# These authors contributed equally to this work
1 Hubrecht Institute, Royal Netherlands Academy of Arts and Sciences (KNAW) and University Medical Centre, Utrecht, The Netherlands
2 Department of Cardiology, University Medical Centre, Utrecht, The Netherlands

*Correspondence to:
Eva van Rooij, PhD
Hubrecht Institute, KNAW
Uppsalalaan 8, 3584 CT Utrecht, The Netherlands.
Phone: +31 30 2121956
e.vanrooij@hubrecht.eu

Supplementary Figure 1

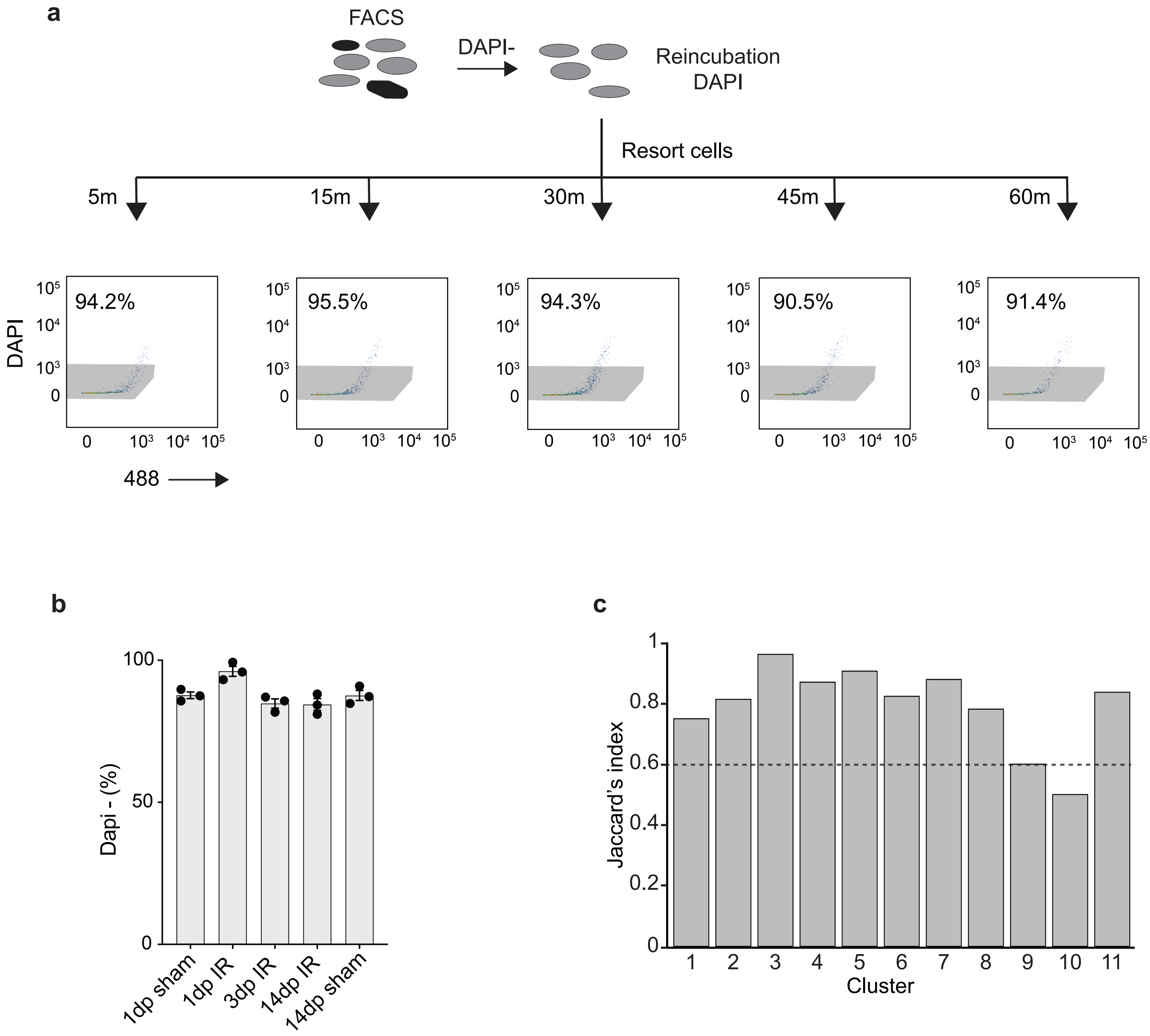

Supplementary Figure 1. **Cell sorting strategy yields viable cells with good membrane integrity from the adult heart. a**, Percentage of DAPI negative cells after sorting and re-incubation with DAPI for several time points. **b**, The fraction of Dapi negative cells as marker of viable cells for all different time points and conditions. Data are presented as mean +/- SEM. **c**, Downstream analysis of sorted cells resulted in robust clustering shown by a bar graph depicting the Jaccard's similarity score for each cluster. As a rule of thumb, each cluster should have an index of > 0.6 to be robust. +/- SEM.

Supplementary Figure 2

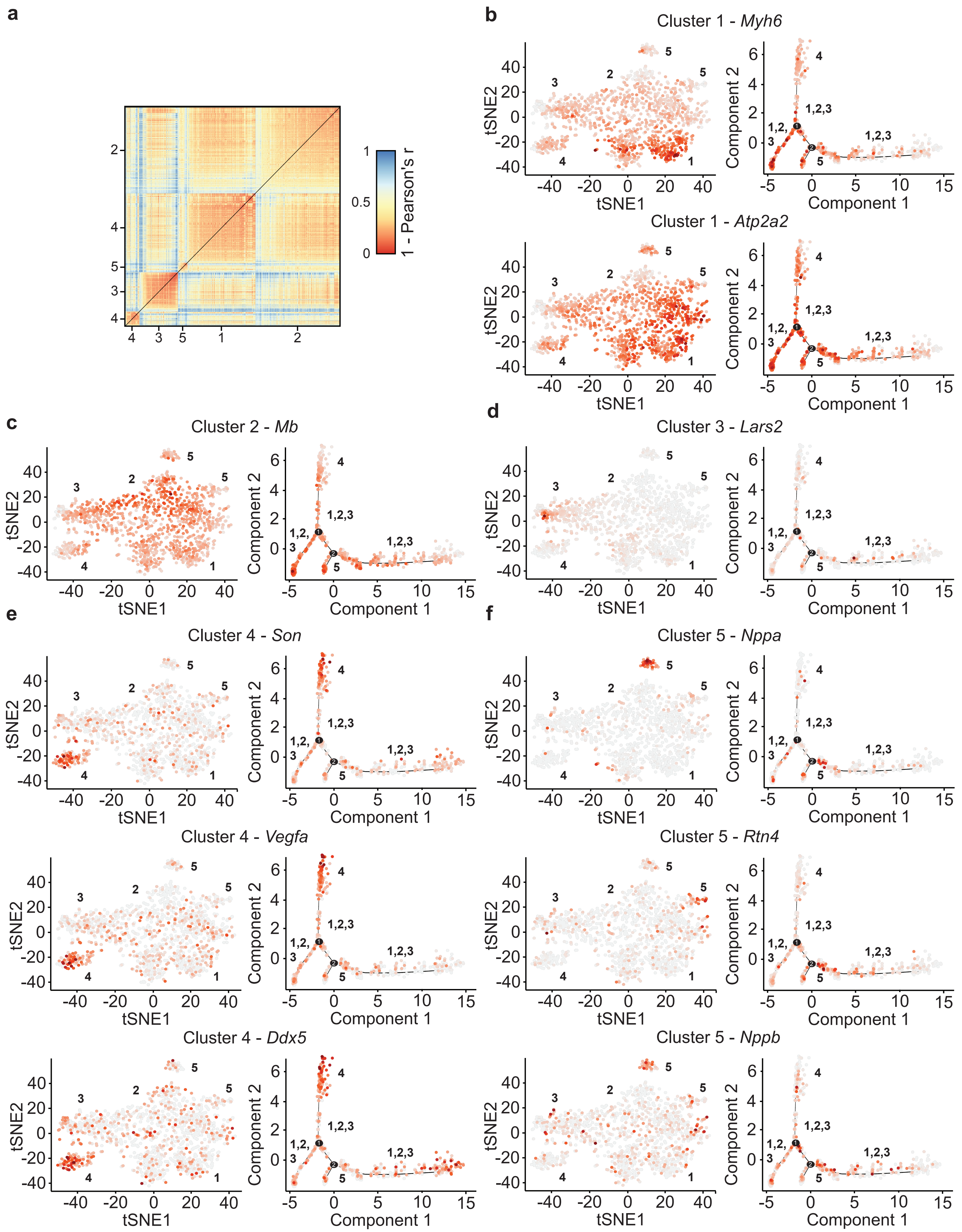

Supplementary Figure 2. tSNE analysis across cardiomyocyte populations indicate gradients of cardiomyocyte marker gene expression. a, Heatmap of the cell-to-cell transcriptome similarities (1-Pearson's correlation coefficient) of 1033 cardiomyocytes obtained from all conditions combined. Cells are clustered based on transcriptome similarity using k-medoids clustering. Clusters identified through this method are also used in fig. 2. **b-f**, tSNE (left) and cell trajectory analysis (right) showing the distribution of expression of genes enriched in cluster 1 (**b**), cluster 2 (**c**), cluster 3 (**d**) cluster 4 (**e**) and cluster 5 (**f**). Expression is shown as normalized read count on a color-coded scale, with red depicting the highest expression and white lowest. Locations in the tSNE plot or in the trajectory plot that contain mostly cells from a cluster are highlighted by numbers of that respective cluster.

Supplementary Figure 3

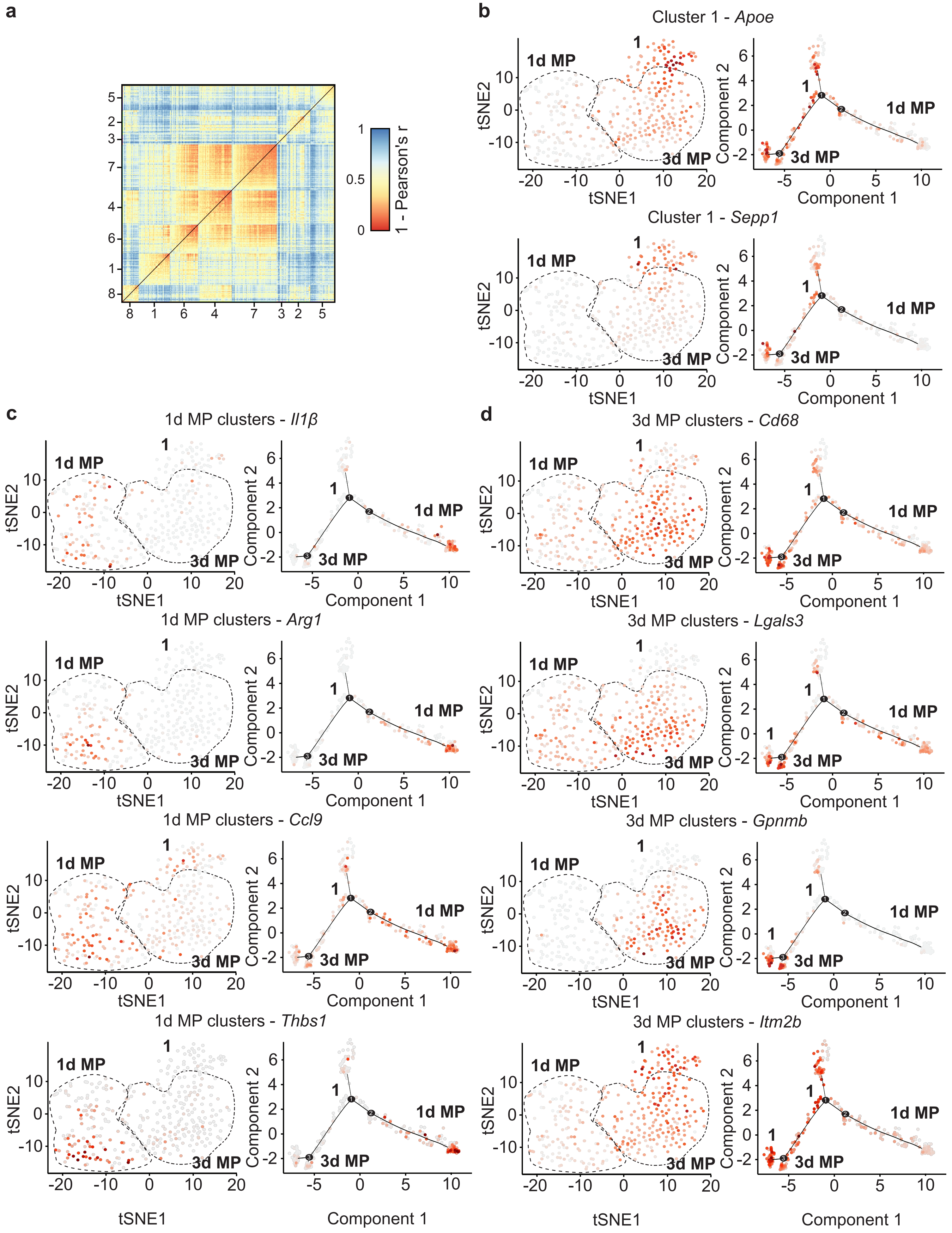

Supplementary Figure 3. tSNE and cell trajectory analysis of macrophages show gradients of expression of cluster-enriched genes, rather than well-defined expression. a, Heatmap of the cell-to-cell transcriptome similarities (1- Pearson's correlation coefficient) of 355 macrophages obtained from all conditions combined. Cells are clustered based on transcriptome similarity using k-medoids clustering. Clusters identified through this method are also used in Fig 3. **b-d**, tSNE (left) and cell trajectory analysis (right) showing the distribution of expression of genes enriched in cluster 1 (**b**), or in clusters enriched in 1dp IR (**c**) or 3dp IR (**d**) macrophages. Expression is shown as normalized read count on a color-coded scale, with red depicting the highest expression and white lowest. Locations in the tSNE plot or branches in the trajectory plot that contain mostly cells from cluster 1 or from the 1dp/3dp IR clusters are highlighted by a number or text, respectively.

Supplementary Figure 4

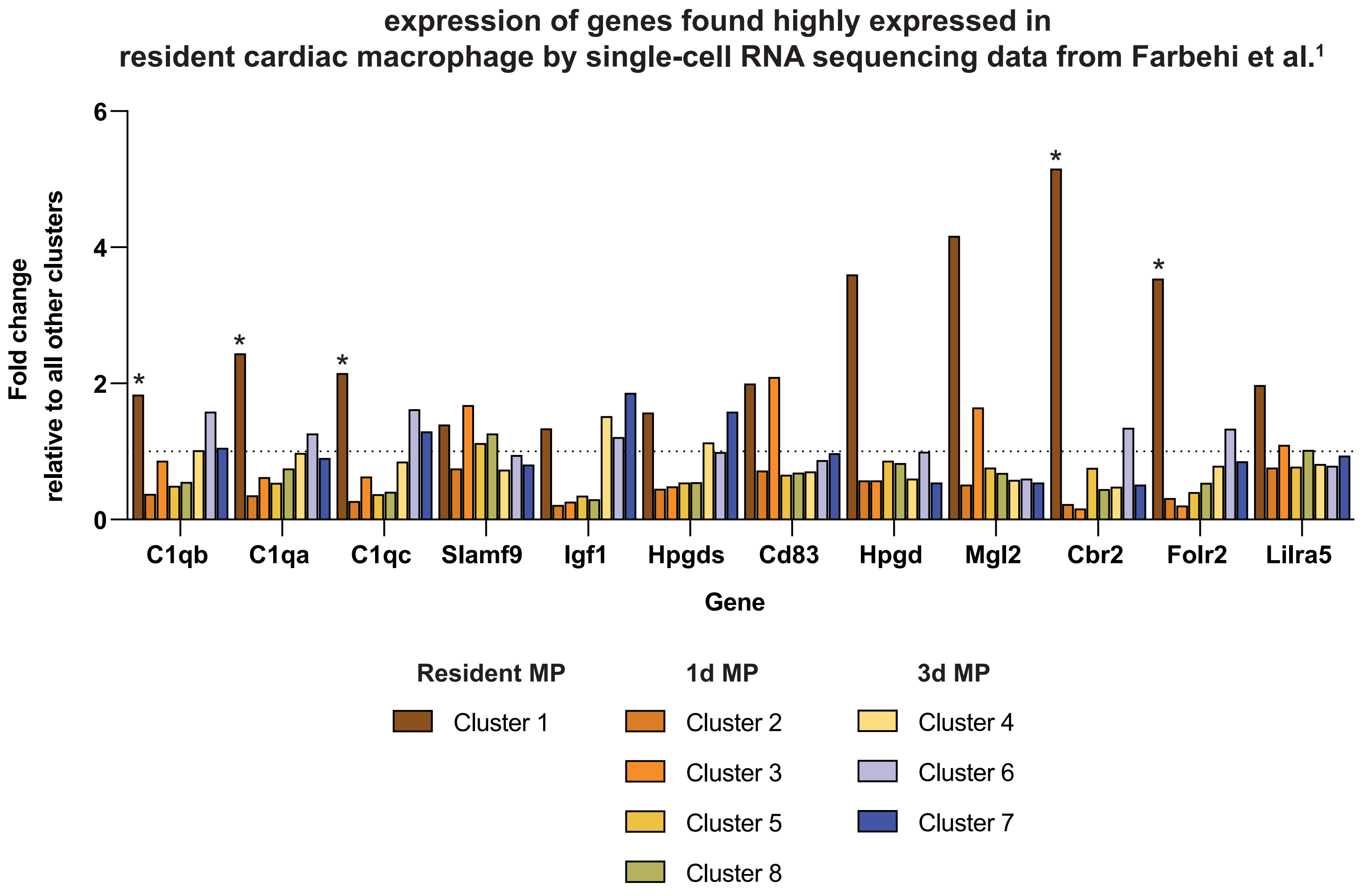

Supplementary Figure 4. Bar graph showing fold change of genes previously shown to be enriched in resident cardiac macrophages by single-cell RNA sequencing. Bar graph depicting the fold change of genes in each macrophage cluster compared to all other clusters. Gene were previously found to be enriched in resident cardiac macrophages in a single-cell RNA sequening study by Ferbahi et al.27. *P<0.05, calculated using binomial counting statistics of the RaceID2 algorithm.

Supplementary Figure 5

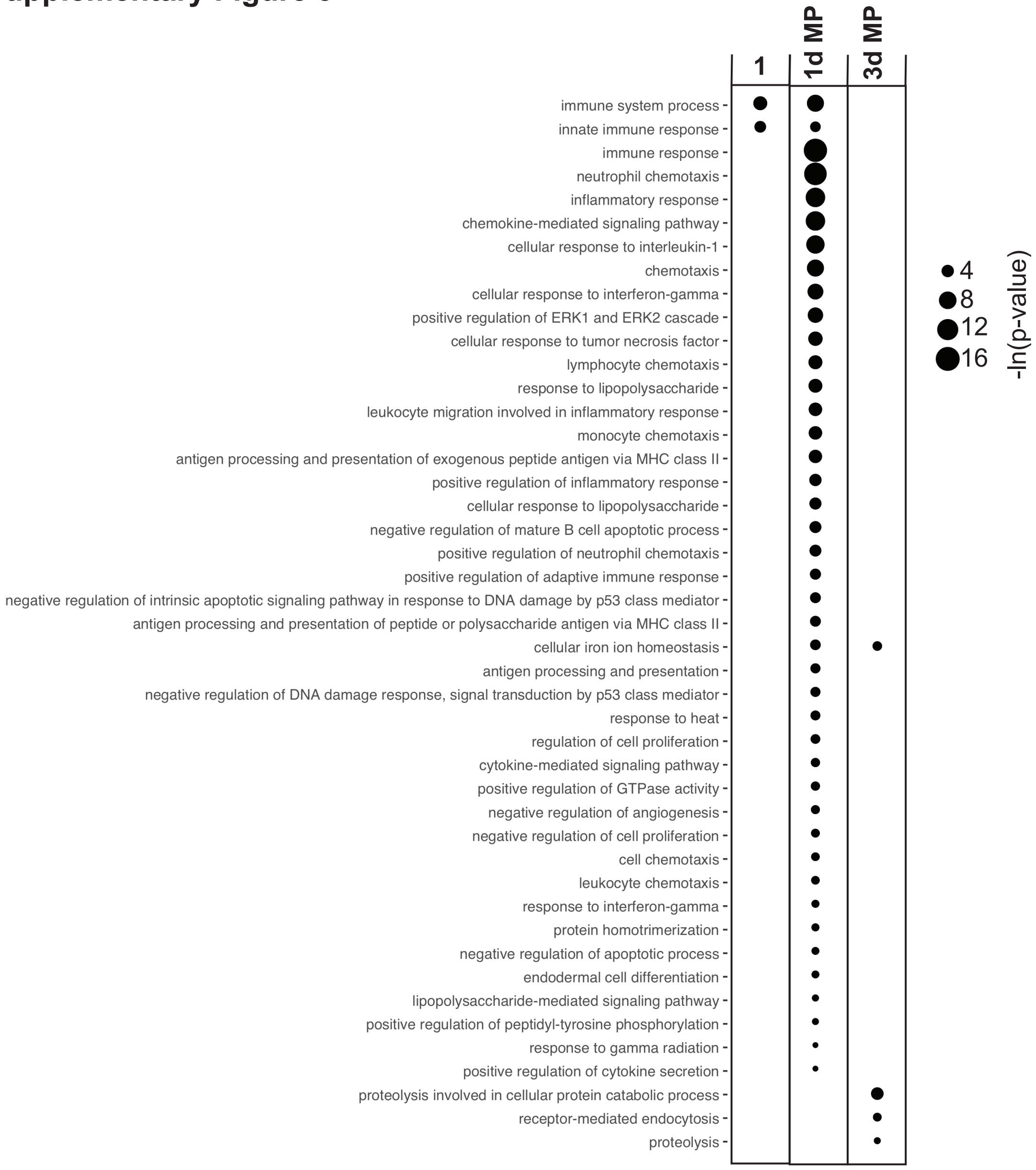

Supplementary Figure 5. Bubble plot of full gene ontology analysis on genes significantly enriched in macrophage clusters (Extended list of Figure 3d).

Supplementary Figure 6

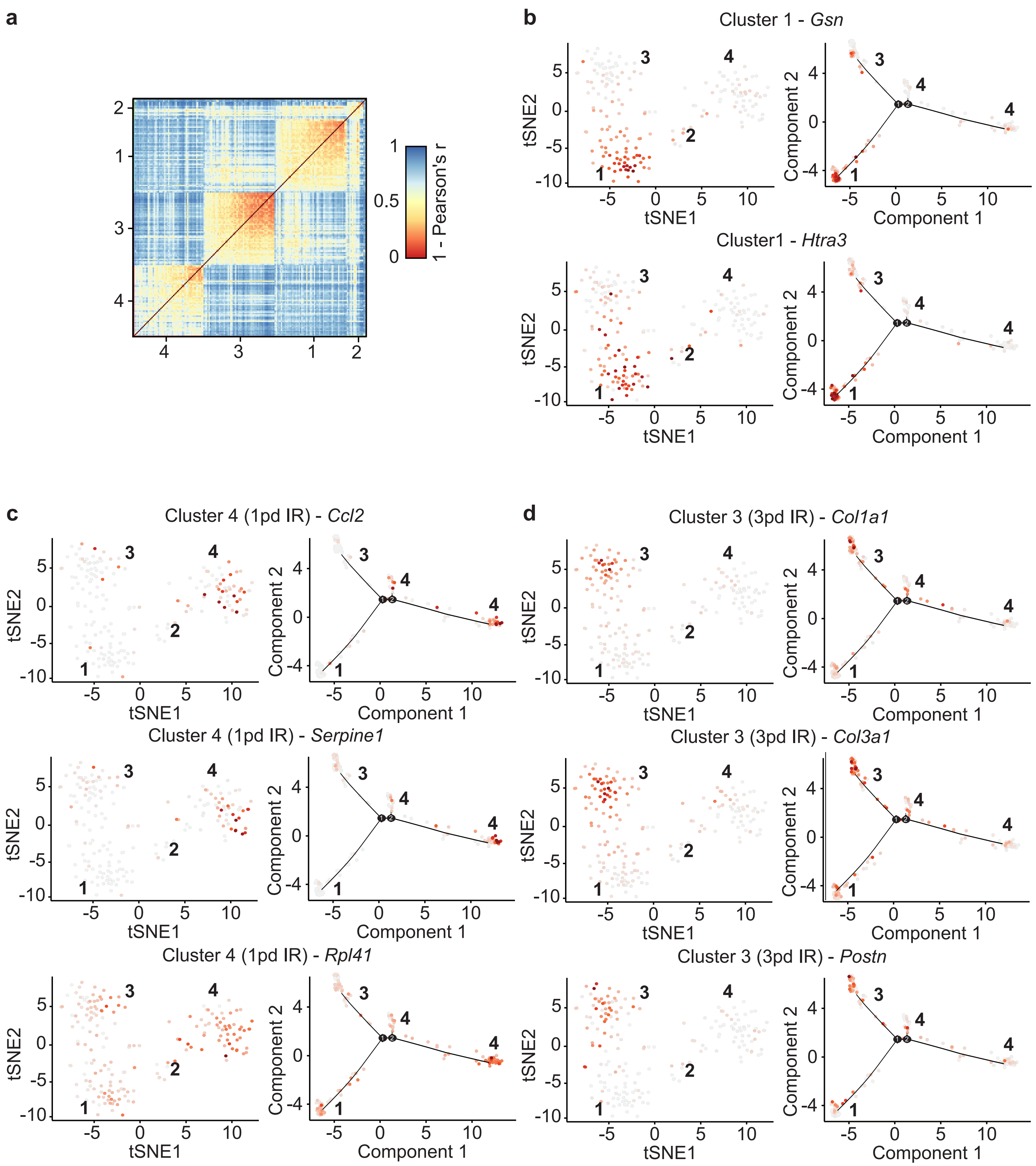

Supplementary Figure 6. tSNE and cell trajectory analysis of fibroblasts with expression of cluster-enriched genes as color coded. a, Heatmap of the cell-to-cell transcriptome similarities (1-Pearson's correlation coefficient) of 184 fibroblasts obtained from all conditions combined. Cells are clustered based on transcriptome similarity using k-medoids clustering. Clusters identified through this method are also used in Fig 4. **b-d**, tSNE (left) and cell trajectory analysis (right) showing the distribution of expression of genes enriched in cluster 1 (**b**), cluster 4 (**c**) and cluster 3 (**d**). Expression is shown as normalized read count on a color-coded scale, with red depicting the highest expression and white lowest. Locations in the tSNE plot or branches in the trajectory plot that contain mostly cells from cluster 1, 3 or 4 are highlighted by number.

Supplementary Figure 7

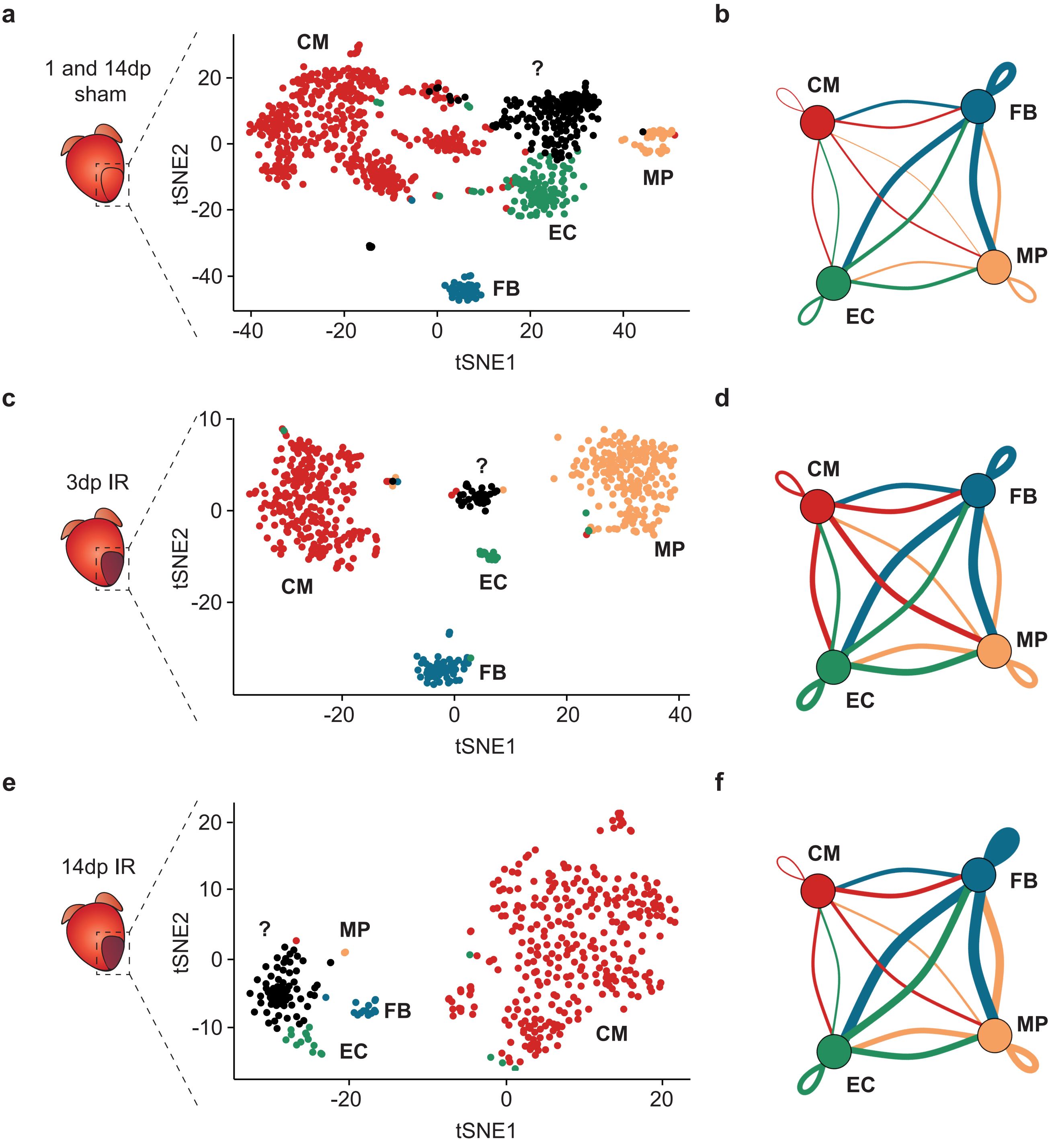

Supplementary Figure 7. Extensive intercellular communication network during different phases of the repair process following ischemia-reperfusion injury. a,c,e, tSNE plot of all cells obtained from 1 and 14dp sham combined (**a**), 3dp IR (**c**) or 14dp IR (**e**). Colors indicate different cell types. CM; cardiomyocytes, FB; fibroblasts, MP; macrophages EC; endothelial cells. **b,d,f,** Spider graph illustrating the potential intercellular communications between cell types by ligand-receptor signaling at 1dp and 14dp sham combined (**b**), 3dp IR (**d**) or 14dp IR (**f**). The line color depicts ligand expression in the cell type with a similar color. Lines connect to cell types that express the corresponding receptor. Line thickness is proportional to the number of ligands expressed in one population for which the receptor is expressed in the other cell type, with loop indicating autocrine signaling.

Supplementary Figure 8

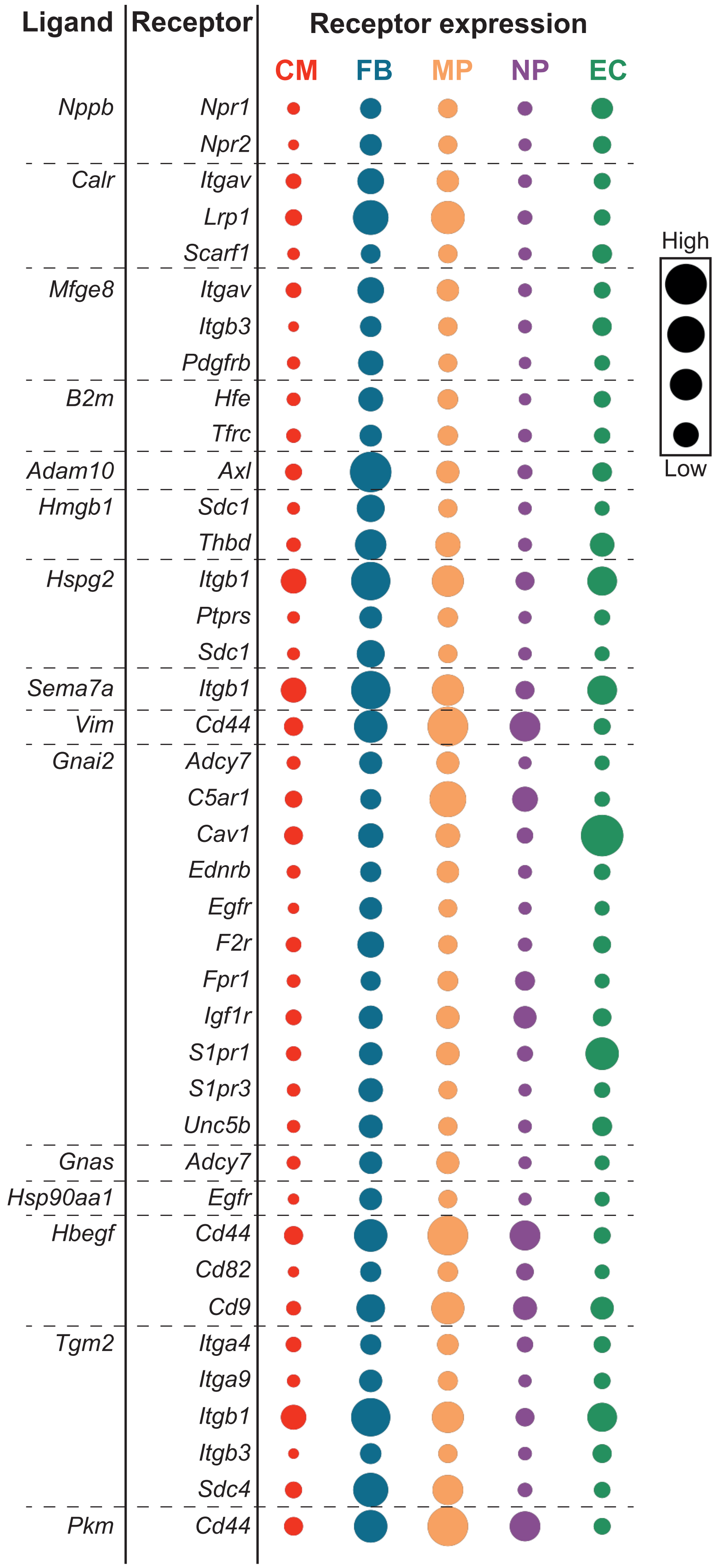

Supplementary Figure 8. Bubble plot showing all ligands upregulated in 1dp IR cardiomyocytes with their cognate receptor(s). The size of the bubble represents expression of each receptor across all identified cardiac cell types 1dp IR. (Extended plot of Figure 5e).

Supplementary Figure 9

a

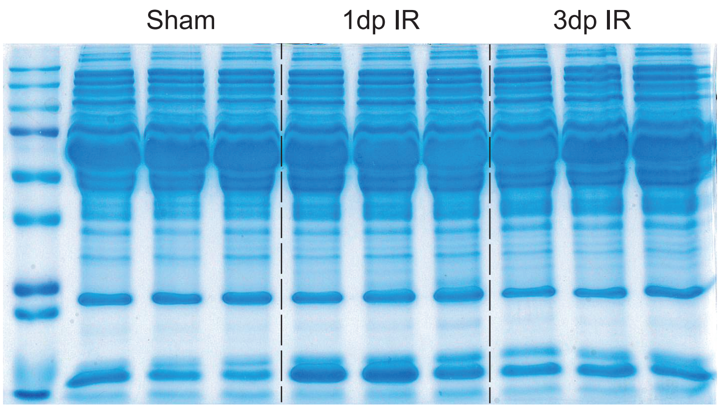

b

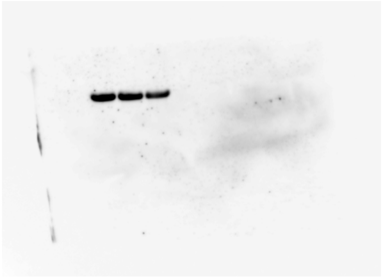

c

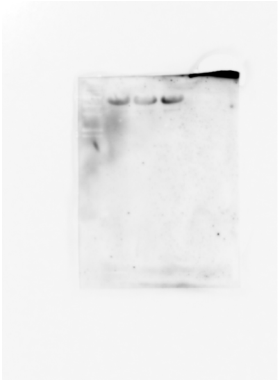

d

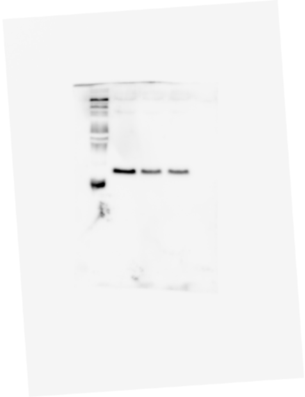

e

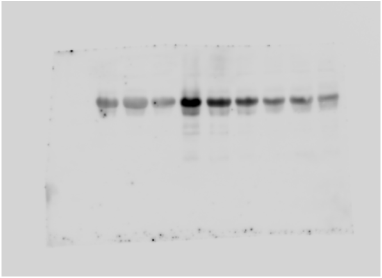

f

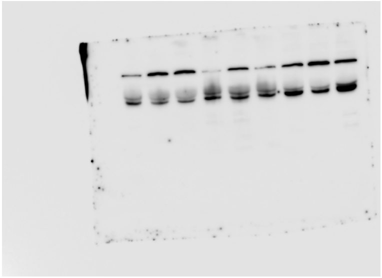

g

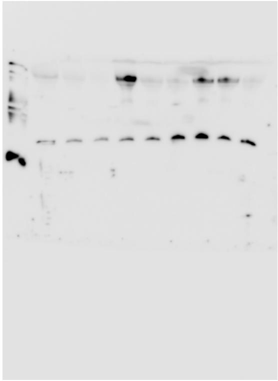

h

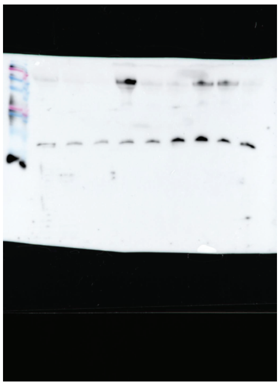

Supplementary Figure 9. Loading control and full uncropped images of Western Blots. a, Coomassie staining confirming equal loading of Western blots used to detect plasma proteins (Fig. 6a). **b-d,** Full uncropped pictures used for the Western Blot images of CALR, MFGE8 and B2m shown in Supplementary Fig 10, respectively. **e-g,** Full uncropped pictures of the Western Blot images of CALR, MFGE8 and B2m shown in Figure 6, respectively. **g**, Example of protein size ladder (corresponding to panel **e**), as black and white image files were used for final images and protein size ladder images were saved seperately.

Supplementary Figure 10

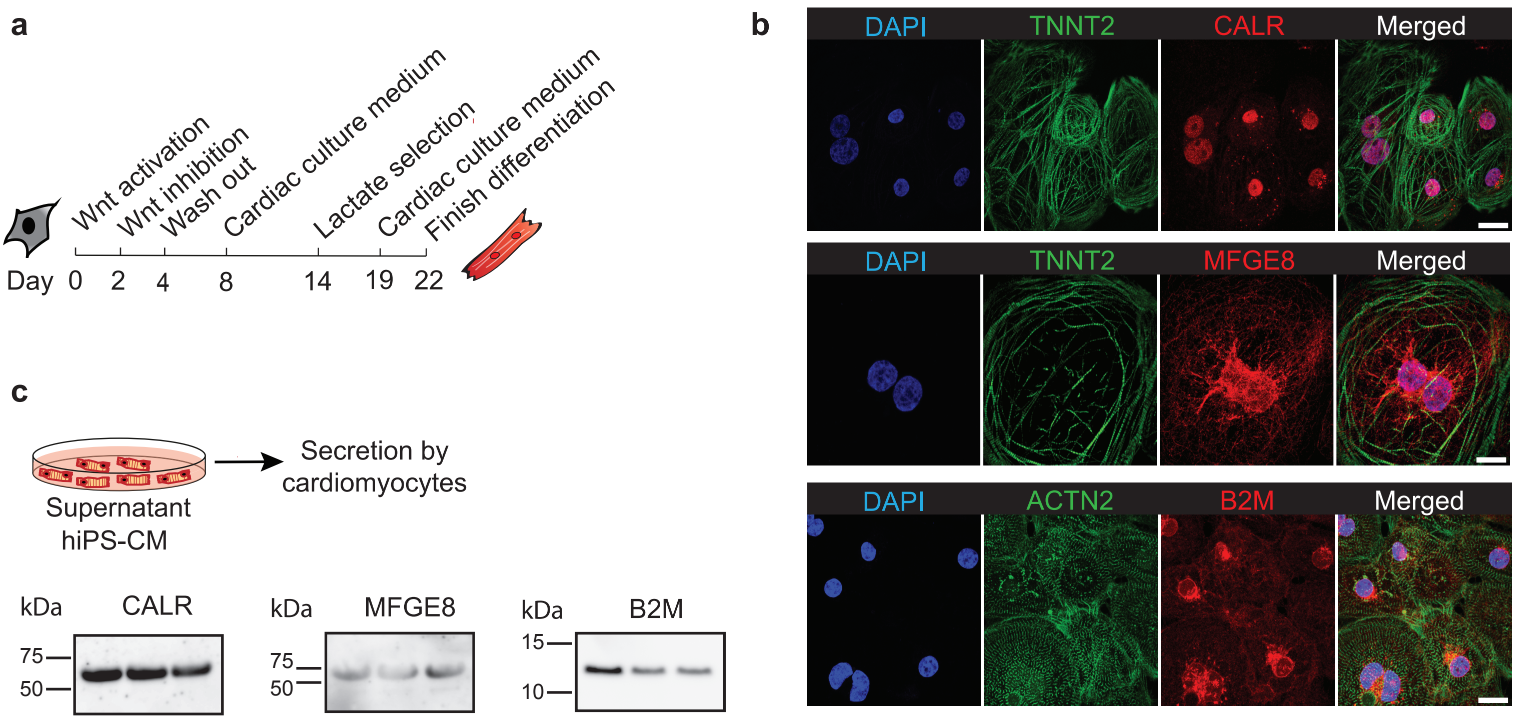

Supplementary Figure 10. Protein expression of CALR, MFGE8 and B2M by human iPS-derived cardiomyocytes. a, Schematic display of the differentiation protocol to derive cardiomyocytes from human iPS cells. **b**, Images captured by fluorescent microscopy showing the expression of 3 selected factors (CALR, MFGE8 and B2M) by human cardiomyocytes derive from iPS cells. Cardiac markers used are cardiac troponin (CTNT) and alpha-Actinin-2 (ACTN2). Scale bar, 20μm. **c**, Western blot on supernatant of human cardiomyocytes obtained from iPS cells showing secretion of CALR, MFGE8 and B2M.

Supplementary Figure 11

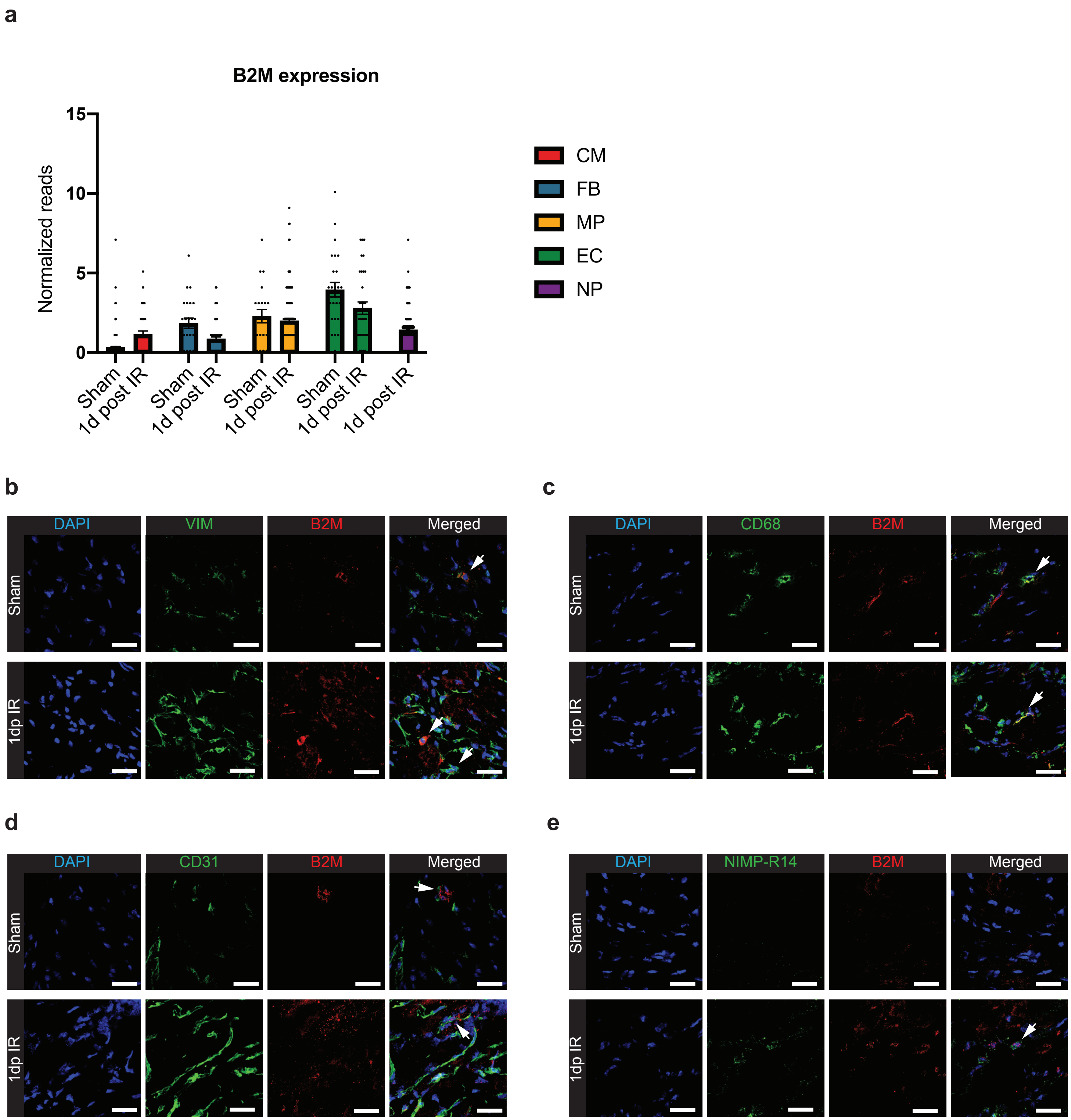

Supplementary Figure 11. B2M expression per cell type. a, Normalized B2m reads per cell type. CM; cardiomyocytes, FB; fibroblasts, MP; macrophages EC; endothelial cells, NP; neutrophils. **b-e**, Images captured by fluorescent microscopy showing expression of B2M by non-cardiomyocyte cells in the heart. All 1dp IR images were taken from the border zone. Cell markers used were Vimentin (FB) (**b**), CD68 (MP) (**c**), CD31 (EC) (**d**), NIMP-R14 (NP) (**e**). Scale bar, 25μm. white arrows indicate cells that show expression of B2M and expression of the corresponding cell markers. Data are presented as mean +/- SEM.

Supplementary Figure 12

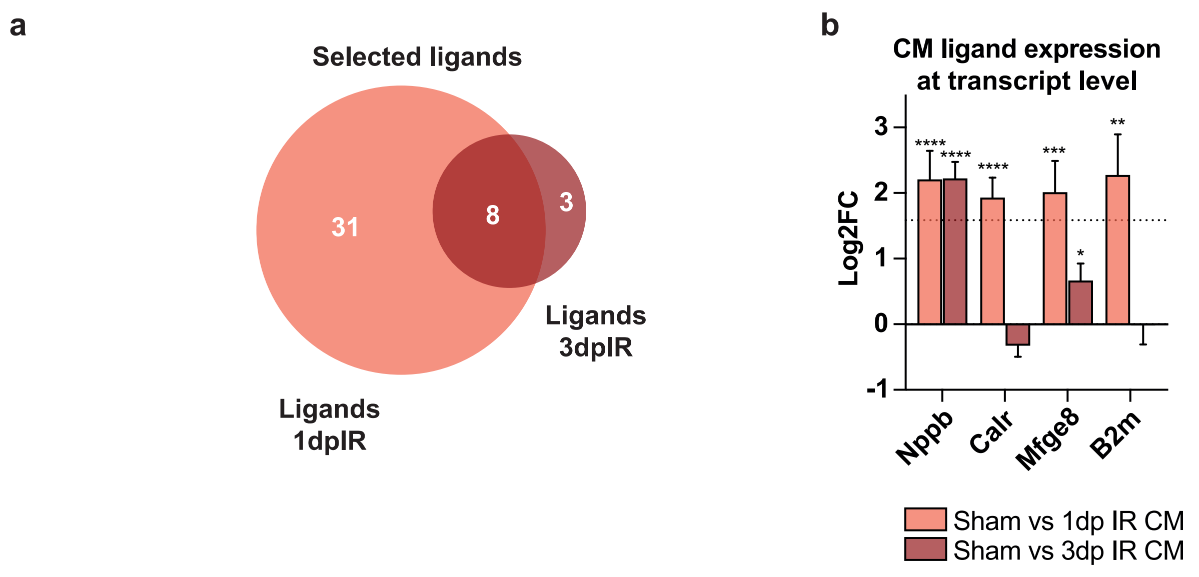

Supplementary Figure 12. Expression of ligands in 1dp IR vs 3dp IR cardiomyocytes. a, Venn-diagram of all ligands that are selected in 1dp IR and 3dp IR cardiomyocytes using the filtering strategy described in Fig. 5A, but before exclusion of candidates that were part of the ECM of highly expressed in other cell types. **b,** Changes in expression of the 3 ligands that were further studied in Fig 5 and Fig 6 in 1dp IR and 3dp IR cardiomyocytes relative to sham cardiomyocytes. Data is shown and the log2 of the fold change (Log2FC) relative to sham cardiomyocytes. The dotted line represents an upregulation of a fold change of 3, which is required for inclusion of the ligand into the screening strategy. Data are presented as mean +/- SEM. * P<0.05, ** P<0.01, *** P<0.001, **** P<0.0001 compared to sham. Log2FC, SEM and p-values are derived from DESeq2 which uses the Wald test with Benjamini-Hochberg correction.

Supplementary references

1. Farbehi, N. et al. Single-cell expression profiling reveals dynamic flux of cardiac stromal, vascular and immune cells in health and injury. Elife 8, doi:10.7554/eLife.43882 (2019).
