# Supplementary material for: Single-cell transcriptomics following ischemic injury identifies a role for B2M in cardiac repair
Source: Commun Biol. 2021 Jan 29;4:146. doi: 10.1038/s42003-020-01636-3 (PMC7846780; doi:10.1038/s42003-020-01636-3)
Supplement: Supplementary file 3 — Description of Additional Supplementary Files [file 42003_2020_1636_MOESM3_ESM.pdf]

Description of Additional Supplementary Files

File name: Supplementary Data 1

Description: Cluster gene profile all cells from all conditions.

File name: Supplementary Data 2

Description: Cluster gene profile cardiomyocytes from all conditions.

File name: Supplementary Data 3

Description: Cluster gene profile macrophages from all conditions.

File name: Supplementary Data 4

Description: Cluster gene profile fibroblast from all conditions.

File name: Supplementary Data 5

Description: Cluster gene profile all cells 1dp IR.

File name: Supplementary Data 6

Description: Cluster gene profile all cells 1dp and 14dp sham.

File name: Supplementary Data 7

Description: Cluster gene profile all cells 3dp IR.

File name: Supplementary Data 8

Description: Cluster gene profile all cells 14dp IR.

File name: Supplementary Data 9

Description: Intercellular communication per condition.

File name: Supplementary Data 10

Description: Source data.
