# Supplementary material for: Single-cell transcriptomics following ischemic injury identifies a role for B2M in cardiac repair
Source: Commun Biol. 2021 Jan 29;4:146. doi: 10.1038/s42003-020-01636-3 (PMC7846780; doi:10.1038/s42003-020-01636-3)
Supplement: Supplementary file 14 — Reporting Summary [file 42003_2020_1636_MOESM14_ESM.pdf]

nature research

Corresponding author(s): E. van Rooij

Last updated by author(s): Dec 16, 2020

Reporting Summary

Nature Research wishes to improve the reproducibility of the work that we publish. This form provides structure for consistency and transparency in reporting. For further information on Nature Research policies, see Authors & Referees and the Editorial Policy Checklist.

Statistics

For all statistical analyses, confirm that the following items are present in the figure legend, table legend, main text, or Methods section.

n/a	Confirmed	
☐	☒	The exact sample size (*n*) for each experimental group/condition, given as a discrete number and unit of measurement
☐	☒	A statement on whether measurements were taken from distinct samples or whether the same sample was measured repeatedly
☐	☒	The statistical test(s) used AND whether they are one- or two-sided<br>*Only common tests should be described solely by name; describe more complex techniques in the Methods section.*
☒	☐	A description of all covariates tested
☐	☒	A description of any assumptions or corrections, such as tests of normality and adjustment for multiple comparisons
☐	☒	A full description of the statistical parameters including central tendency (e.g. means) or other basic estimates (e.g. regression coefficient) AND variation (e.g. standard deviation) or associated estimates of uncertainty (e.g. confidence intervals)
☐	☒	For null hypothesis testing, the test statistic (e.g. *F*, *t*, *r*) with confidence intervals, effect sizes, degrees of freedom and *P* value noted<br>*Give P values as exact values whenever suitable.*
☒	☐	For Bayesian analysis, information on the choice of priors and Markov chain Monte Carlo settings
☒	☐	For hierarchical and complex designs, identification of the appropriate level for tests and full reporting of outcomes
☒	☐	Estimates of effect sizes (e.g. Cohen's *d*, Pearson's *r*), indicating how they were calculated

Our web collection on statistics for biologists contains articles on many of the points above.

Software and code

Policy information about availability of computer code

Data collection	See methods
Data analysis	See methods

For manuscripts utilizing custom algorithms or software that are central to the research but not yet described in published literature, software must be made available to editors/reviewers. We strongly encourage code deposition in a community repository (e.g. GitHub). See the Nature Research guidelines for submitting code & software for further information.

Data

Policy information about availability of data

All manuscripts must include a data availability statement. This statement should provide the following information, where applicable:

- Accession codes, unique identifiers, or web links for publicly available datasets
- A list of figures that have associated raw data
- A description of any restrictions on data availability

Raw data and processed read count tables are available online at GEO with accession code GSE146285.

Field-specific reporting

Please select the one below that is the best fit for your research. If you are not sure, read the appropriate sections before making your selection.

☒ Life sciences ☐ Behavioural & social sciences ☐ Ecological, evolutionary & environmental sciences

For a reference copy of the document with all sections, see nature.com/documents/nr-reporting-summary-flat.pdf

Life sciences study design

All studies must disclose on these points even when the disclosure is negative.

Sample size	All experiments were performed using triplicates to aim for reproducibility. No statistical methods were used to predetermine sample size.
Data exclusions	No data was excluded
Replication	All experiments were performed using at least two independent replicates. All findings were replicated.
Randomization	For IR experiments, all animals were randomly assigned to IR or sham treatment.
Blinding	Researchers were not blinded in the study

Reporting for specific materials, systems and methods

We require information from authors about some types of materials, experimental systems and methods used in many studies. Here, indicate whether each material, system or method listed is relevant to your study. If you are not sure if a list item applies to your research, read the appropriate section before selecting a response.

Materials & experimental systems

n/a	Involved in the study	
☐	☒	Antibodies
☐	☒	Eukaryotic cell lines
☒	☐	Palaeontology
☐	☒	Animals and other organisms
☒	☐	Human research participants
☒	☐	Clinical data

Methods

n/a	Involved in the study	
☒	☐	ChIP-seq
☐	☒	Flow cytometry
☒	☐	MRI-based neuroimaging

Antibodies

Antibodies used	See methods sections 'Histology and immunofluorescence' and 'Western blot'
Validation	All antibodies were previously published and showed expected protein weight or anatomical and/or cell type specific expression.

Eukaryotic cell lines

Policy information about cell lines

Cell line source(s)	NIH/3T3 cells were obtained from ATCC (ATCC CRL-1658).
Authentication	NIH/3T3 cells were authenticated based on morphology and expression of fibroblast marker genes col1a1, col1a2
Mycoplasma contamination	We confirm that NIH/3T3 cells tested negative for mycoplasma contamination
Commonly misidentified lines (See ICLAC register)	None

Animals and other organisms

Policy information about studies involving animals; ARRIVE guidelines recommended for reporting animal research

Laboratory animals	All animals used were of C57BL/6J background and 8 weeks old males.
Wild animals	This study did not involve wild animals
Field-collected samples	This study did not involve samples collected from the field
Ethics oversight	All animal studies were performed in accordance with institutional guidelines and with approval of the Animal Welfare Committee of the Royal Netherlands Academy of Arts and Sciences.

Note that full information on the approval of the study protocol must also be provided in the manuscript.

Flow Cytometry

Plots

Confirm that:

☒ The axis labels state the marker and fluorochrome used (e.g. CD4-FITC).

☒ The axis scales are clearly visible. Include numbers along axes only for bottom left plot of group (a 'group' is an analysis of identical markers).

☒ All plots are contour plots with outliers or pseudocolor plots.

☒ A numerical value for number of cells or percentage (with statistics) is provided.

Methodology

Sample preparation	The process of sample preparation is described in detail in the methods section 'Cardiac tissue dissociation into single-cell suspension.'
Instrument	FACSAriaII (BD bioscience)
Software	Analysis was performed using FlowJo v10.6.0
Cell population abundance	Abundance of different cell populations in the post-sorted cells which passed quality control as described in the methods are depicted in figure 1.
Gating strategy	Gating strategy is described in the method section 'Flow cytometry and cell viability/RNA quality control'

☐ Tick this box to confirm that a figure exemplifying the gating strategy is provided in the Supplementary Information.
